# Real-Time Non-Invasive Monitoring of Short-Chain Fatty Acids in Exhaled Breath

**DOI:** 10.3389/fchem.2022.853541

**Published:** 2022-04-26

**Authors:** Joris Meurs, Evangelia Sakkoula, Simona M. Cristescu

**Affiliations:** Exhaled Biomarkers and Exposure Group, Department of Analytical Chemistry & Chemometrics, Institute for Molecules and Materials, Radboud University, Nijmegen, Netherlands

**Keywords:** short-chain fatly acids, exhaled breath, gut micobiome, SRI/PTR-ToF-MS, non-invasive monitoring

## Abstract

Short-chain fatty acids (SCFAs) are important metabolites produced by the gut microbiome as a result of the fermentation of non-digestible polysaccharides. The most abundant SCFAs are acetic acid, propionic acid, and butyric acid which make up 95% of this group of metabolites in the gut. Whilst conventional analysis SCFAs is done using either blood or fecal samples, SCFAs can also be detected in exhaled breath using proton transfer reaction-time-of-flight- mass spectrometry (PTR-ToF-MS) using H_3_O^+^ for ionization. However, no investigation has been performed to characterize the reactions of SCFAs with H_3_O^+^ and with other reagent ions, such as O_2_^+^ and NO^+^. Gas-phase samples of acetic acid, propionic acid, and butyric acid were analyzed with SRI/PTR-ToF-MS under dry and humid conditions. The ions generated and their distribution was determined for each reagent ion. It was found the humidity did not influence the product ion distribution for each SCFA. Using H_3_O^+^ as a reagent ion, SRI/PTR-ToF-MS analysis of an exhaled breath sample was performed in real-time to demonstrate the methodology. The presence of SCFAs in exhaled breath was confirmed by thermal desorption—gas chromatography—mass spectrometry (TD-GC-MS). Breath sampling repeatability was within acceptable limits (<15%) for an analytical methodology for each investigated SCFA. Nutritional intervention studies could potentially benefit from real-time monitoring of exhaled SCFAs as an alternative to measuring SCFAs invasively in blood or fecal samples since it is non-invasive, and requires minimal time investment from participants.

## Introduction

Dietary intake has an enormous impact on human health (Singh et al., 2017). At a young age, low dietary quality can have adverse effects on cognitive development (Bellisle, 2004; Miquel et al., 2018); therefore, proper choice of food intake is essential to prevent conditions like obesity, cardiovascular disease, and cognitive decline (Bellisle, 2004; Miquel et al., 2018).

Alteration in food consumption leads to changes in gut microbiome activity (Johnson et al., 2020). As such, the microbiome releases by-products and secondary metabolites from dietary components that can serve as an excellent indicator for assessing diet-induced metabolic changes. Among these metabolites is the group of short-chain fatty acids (SCFAs) from which acetic, propionic and butyric acid make up 95% of the SCFAs produced in the gut (Wu et al., 2021). Production of SCFAs is a result of fermentation of (partially) non-digestible polysaccharides (NDP) (Forkman, 2009) in the colon, mainly emanating from highly resistant starches (Cummings et al., 1986). Monitoring SCFAs could therefore potentially provide information on the quality (fiber content) of an individual’s diet (Ou et al., 2013). Currently, analysis of SCFAs is in general performed using either blood or fecal samples followed by chromatography and subsequent mass spectrometry detection (van Eijk et al., 2009; Chan et al., 2017; Chen et al., 2021). These methods are however labor-intensive and require (partially) invasive sample collection. Therefore, it is not suitable to track SCFAs concentrations in real-time.

Previous studies reported detection of SCFAs in gas-phase samples using proton transfer reaction-time-of-flight-mass spectrometry (PTR-ToF-MS) (Hartungen et al., 2004; Henderson et al., 2021). This is a soft ionization technique in which volatile organic compounds (VOCs) with a higher proton affinity than water become ionized as a result of proton transfer in the gas phase (Lindinger et al., 1998a). Von Hartungen *et al.* showed that SCFA could be analyzed from axillary samples. More recently, Henderson *et al.* used PTR-ToF-MS to non-invasively monitor in real-time SCFAs in breath as potential indicators of gut microbiota activity relating to exercise and medication use. It was found that the SCFA concentration in exhaled breath increased throughout prolonged exercise (Henderson et al., 2021). In addition, butyric acid was identified as a potential non-invasive marker for exercise-induced inflammation (Henderson et al., 2022).

Technical advances in the PTR-ToF-MS include the use of different selective reagent ions (SRI) such as H_3_O^+^ (most common), NO^+^, O_2_^+^, etc. for ionizing the compounds of interest. This feature allows detection of a broader range of analytes, a better isomeric separation for several classes of organic chemical compounds, and an improved level of selectivity (Jordan et al., 2009b).

Whilst PTR-ToF-MS shows the great potential for real-time monitoring of the most abundant SCFAs (acetic acid, propionic acid, and butyric acid), the conditions for their optimal analysis in the exhaled breath have not yet been investigated. Here, we used SRI/PTR-ToF-MS to optimize and validate a method for SCFAs quantification in exhaled breath. For this, we studied the differences in ion branching when different reagent ions (H_3_O^+^, NO^+^, and O_2_^+^) when various drift tube reduced field energies (*E*/*N*) were used. The branching ratios were determined under dry and humid conditions, respectively. Using the optimum parameters, the method will be demonstrated for real-time measurement of SCFAs in exhaled breath.

## Materials and Methods

### Chemicals

Acetic acid (>99%) was purchased from Sigma-Aldrich (Zwijndrecht, the Netherlands). Propionic acid (99%+) and butyric acid (99%+) were obtained from Alfa Aesar (Karlsruhe, Germany). All chemical standards were in the liquid phase.

### Sample Preparation

It has been reported that humidity might affect the ion intensities in PTR-(ToF)-MS (Trefz et al., 2018). Therefore, it is of the essence for method optimization to understand what effect humidity has on the product ion intensities for each SCFA.

Gas standards for *E/N* optimization were generated in a similar way as described by Malásková *et al* (Malásková et al., 2019a). 10-mL headspace vials were filled with 1 mL of the liquid chemical standard and left to equilibrate overnight at room temperature.

To measure under dry (normal) conditions, Tedlar^®^ bags were filled with 2 L synthetic air (5.5, Linde Gas, Dieren, the Netherlands). From each headspace vial, a set volume of headspace was aspirated depending on the volatility of the chemical standard (acetic acid: 0.2 mL; propionic acid: 0.5 mL; butyric acid: 1.0 mL; isobutyric acid: 1.0 mL; ethyl acetate: 0.1 mL). The collected headspace was transferred to a Tedlar^®^ bag filled with synthetic air.

For measurements under humid conditions, such as exhaled breath (estimated 100% relative humidity), the synthetic airflow was first led through a bottle containing deionized water. The humidified air was subsequently collected in a Tedlar^®^ bag (total volume of 2 L). Afterward, the headspace was sampled from the vials containing the liquid chemical standards and injected into the Tedlar^®^ bag *via* the septum.

Before and during analysis, Tedlar^®^ bags were kept in an oven at 37°C. The Tedlar^®^ bags were then connected to an SRI/PTR-ToF-MS 8000 (Ionicon Analytik GmbH, Innsbruck, Austria) *via* a heated 1/16’’ polyether ether ketone (PEEK) tubing (T = 80°C).

### *E/N* Optimization of SRI/PTR-ToF-MS Parameters for Measurement of SCFAs

The principle of PTR-ToF-MS has been described in detail elsewhere (Lindinger et al., 1998a; Lindinger et al., 1998b). Briefly, H_3_O^+^(H_2_O)_*n*_ (*n* = 0, 1, 2, … ) is generated through a hollow cathode discharge of water vapor. These reagent ions are subsequently transferred to a drift tube, where also the analytes are injected. Proton transfer reaction from the hydronium ion takes place when the proton affinity of the analyte exceeds the proton affinity of water (691 kJ·mol^−1^). The proton transfer can be both dissociative and non-dissociative. Fragmentation of protonated molecules can occur spontaneously or originate from collision-induced reactions of the reagent ions with (charged) analytes (Malásková et al., 2019b). Therefore, the mass spectrum of a single compound may be composed of the protonated molecule, as well as fragment ions derived from the parent molecule. Throughout all experiments, a PTR-TOF 8000 instrument with SRI option (5000 *Δm/m*) was used (Ionicon Analytik GmbH, Innsbrück, Austria).

The reagent ions NO^+^ and O_2_^+^ were used to investigate the reaction products under various drift tube conditions and their potential to distinguish between isomers without the use of chromatography. SRI/PTR-ToF-MS is employed to explore its potential to discriminate between butyric acid and its isomers isobutyric acid and ethyl acetate.

For all product ion measurements, the drift tube pressure and temperature were kept at 2.3 mbar and 80°C, respectively. The inlet flow was set to 20 sccm. The drift tube voltage was altered from 330 to 915 V to cover a reduced electric field range (*E/N*) from 80 to 210 Td with increments of 10 Td. For each reduced electric field, spectra were acquired with a time resolution of 500 ms for 30 s resulting in a total of 60 spectra per *E/N*. The switching of the drift tube voltage and reagent ion was performed in an automated sequence. Before the start of the automated sequence, the signal was allowed to stabilize for 1 min. The product ion distribution was investigated for ions which account for at least 3% of the total intensity of the product ions (Malásková et al., 2019a).

### Method Validation With Chemical Standards

SCFA standards were diluted to 25 mg·L^−1^ in deionized water. By using the inert gas stripping method, gas standards were generated from the aqueous solutions (Karl et al., 2003). The headspace concentration was calculated using Henry’s law constant. Further dilutions were created using two mass flow controllers (Supplementary Figure S1). The concentrations used were 4, 10, 20, 40, 60, 80, 100 part-per-billion volume (ppbV) for each SCFA. Acetone was used as exhaled breath tracer and was calibrated using a gas cylinder (1 ppm in N_2_, Linde Gas, Dieren, the Netherlands). Concentrations of acetone were measured at 40, 80, 120, 160, and 200 ppbV. The corresponding SRI/PTR-ToF-MS signals were investigated for linearity, limit of detection (LOD), limit of quantification (LOQ), and repeatability. Measurements were done using three repeats. The LOD and LOQ were calculated according to the definition of the International Union of Pure and Applied Chemistry (IUPAC), i.e. the LOD and LOQ are equal to the blank signal plus three and plus ten times the standard deviation of the blank signal, respectively. Linearity was assessed by calculating the coefficient of determination (*R* ^2^).

### Exhaled Breath Collection for Off-Line Measurements

Five volunteers were asked to exhale through a mouthpiece bacterial filter (GVS, Morecambe, United Kingdom) through a one-way breathing tube fitted with Teflon tubing connected to a 3 L Tedlar^®^ sampling bag. Before sampling, participants were asked to rinse their mouths with water. A small discard bag of 150 mL was attached to the start of the sampling line to account for the dead space and thus allowing only the end-tidal breath to be collected in the sampling bag. The exhaled breath samples were then transferred to the sorbent tubes at a flow rate of 80 mL·min^−1^. Samples were collected and analyzed in triplicate. For SRI/PTR-ToF-MS analysis, the Tedlar^®^ bag was directly connected to the PEEK inlet of the instrument. The temperature and flow of the heated inlet line were set to 110°C and 80 sccm. The drift tube was operated at 110°C under 2.3 mbar and at a drift voltage of 500 V. The resulting *E/N* ratio was 130 Td.

### Demonstration of Real-Time Monitoring SCFAs in Exhaled Breath Samples

To demonstrate the suitability of the optimized conditions of the SRI/PTR-ToF-MS for real-time detection of SCFAs in exhaled breath, five healthy volunteers were asked to exhale into the instrument *via* a commercial breath sampler (Loccioni^®^, Angeli di Rosora, Italy). The breath sampling procedure has been validated previously (Henderson et al., 2021). Briefly, the participants were asked to rinse their mouth with water before exhaling through a mouthpiece into a calibrated buffer pipe equipped with a CO_2_-sensor at a constant flow rate of 50 mL·s^−1^. The PEEK inlet tubing of the PTR was directly inserted into the calibrated buffer pipe allowing the collection of breath profiles in real-time. In total, 5 consecutive exhaled breath samples were monitored to check the reproducibility of the sampling. For this, the coefficient of variation (CV%) was calculated (ratio of the standard deviation and the average concentration multiplied by 100%) for the exhaled breath. Between exhalations, the volunteer withdrew from the buffer pipe and the background of room air was recorded (Henderson et al., 2021).

For the SRI/PTR-ToF-MS analysis of exhaled breath, H_3_O^+^ was used as reagent ion. The temperature and flow of the heated inlet line were set to 110°C and 300 sccm. The drift tube was operated at 110°C under 2.3 mbar and at a drift voltage of 500 V. The resulting *E/N* ratio was 130 Td.

### Method Validation With Thermal Desorption—Gas Chromatography—Mass Spectrometry

To confirm the presence of each SCFA in exhaled breath as detected by SRI/PTR-ToF-MS, chemical standards, and exhaled breath was analyzed with TD-GC-MS. First, gas standards from liquid chemical standards were generated as described in *Method Validation with Chemical Standards Section* and were directly collected onto Tenax TA/Carbograph 5TD sorbent tubes. Afterward, the sorbent tubes were dry purged with N_2_ for one minute at a flow rate of 6 L·h^−1^. Analysis of trapped VOCs was then performed using a TD-20 thermal desorption system coupled to a GC-MS (QP2010 Ultra, Shimadzu, Kyoto, Japan). Desorption was carried out at a temperature of 260°C under a flow of helium (60 mL·min^−1^) for 8 min. Desorbed VOC was collected on a focusing trap (Tenax TA; Shimadzu, Kyoto, Japan) which was kept at −20°C. After, the focusing trap was rapidly heated to 250°C for 5 min to release the VOC which were then transferred to a CP-Sil 19 CF capillary column (Agilent Technologies, Amstelveen, the Netherlands). The split ratio was set to 50:1 for the reference standards and 5:1 for exhaled breath samples. The column temperature was initially kept at 40°C for 5 min after which the temperature was increased to 250°C at a rate of 5 °C·min^−1^. The final temperature was kept for 5 min. Mass spectra were acquired in the *m/z* range 30–500 at a scan rate of 11 scans·s^−1^. Using the reference standards, the retention time and diagnostic ions were determined for acetic acid, propionic acid, and butyric acid. Analysis was performed in triplicate.

### Data Analysis

All data processing was carried out in PTR-Viewer (Ionicon Analytik GmbH, Innsbruck, Austria). Mass spectra were calibrated using *m/z* 21.022 (H_3_^18^O^+^) and *m/z* 203.943 (PerMasCAL) for H_3_O^+^, and *m/z* 33.998 (^16^O^18^O^+^) and *m/z* 45.992 (NO_2_^+^) for both O_2_^+^ and NO^+^. Raw counts were used to present the product ion distributions, so results can be compared easily between instruments (Malásková et al., 2019b). For SRI/PTR-ToF-MS analysis of exhaled breath samples with H_3_O^+^, ion counts were normalized against H_3_^18^O^+^ (× 500). Further statistical analysis was done in MATLAB (R2020a, The Mathworks, Inc.). GC-MS data were processed using OpenChrom (v 1.4.0) (Wenig and Odermatt, 2010). Comparison between two groups was done using the Mann-Whitney test. A *p*-value less than 0.05 was considered significant. Quantification results for TD-GC-MS and SRI/PTR-ToF-MS were compared using correlation analysis. Linearity was assessed by fitting the function *y = ax + b* in which *y* is the measured intensity, *x* is the concentration, *a* is the slope, and *b* is the intercept.

The background signal was determined by averaging the signal for each trace (SCFA or acetone) for 1 min. The start and end of the exhalation were defined as consecutive scans with a concentration greater than the average background concentration plus three standard deviations of the background signal. The alveolar breath phase was selected using a breath tracker algorithm (Trefz et al., 2013).

## Results and Discussion

### *E*/*N* Optimization of SCFAs With H_3_O^+^

Acetic acid, propionic acid, and butyric acid were analyzed with PTR-ToF-MS under normal (dry) and humid (exhaled breath) conditions to determine which product ions are formed (ion branching ratios) at different reduced electric fields and investigate whether humidity affects the product ion distribution.

**FIGURE 1:**
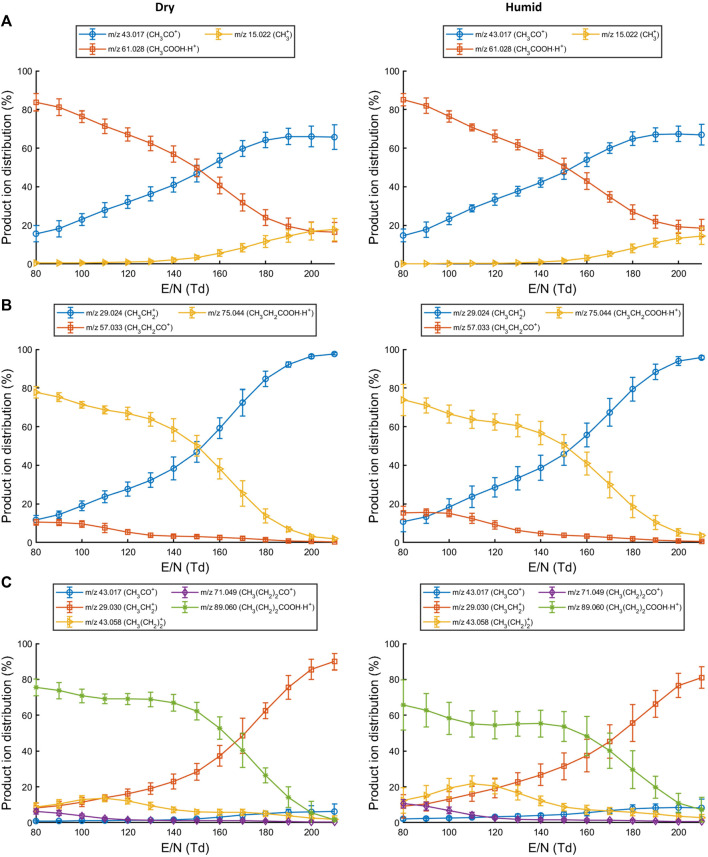
Production ion distributions for reactions of **(A)** acetic acid, **(B)** propionic acid and **(C)** butyric acid with H3O+ in the drift tube at different reduced electric fields. Error bars represent three standard deviations.

At *E/N* of 140 Td or lower, the parent ion (CH_3_COOHH^+^; *m/z* 61.03) is the most abundant for acetic acid (Figure 1A). Increasing the *E/N* to 150 Td or higher led to a substantially increased fragmentation. For acetic acid, the parent ion at *m/z* 61.03 fragments to form CH_3_CO^+^ (*m/z* 43.02) or CH_3_^+^ (*m/z* 15.02) (Table 1). The formation of CH_3_^+^ indicates a neutral loss of HCOOH which was also previously observed when analyzing hexanoic acid (Romano and Hanna, 2018). No difference in product ion distribution was observed as a result of humid drift tube conditions.

**TABLE 1:** Drift tube reactions with H_3_O^+^ and ion branching ratios of SCFAs at selected reduced electric fields under humid conditions.

			Relative Abundance (%)		
SCFA	Reaction Product	m/z	*E/N* 80 Td	*E/N* 140 Td	*E/N* 210 Td
H_3_O^+^					
Acetic acid	CH_3_COOH·H^+^	61.03	88	57	18
	CH_3_CO^+^	43.02	12	42	67
	CH_3_^+^	15.02	—	1	15
Propionic acid	CH_3_CH_2_COOH·H^+^	75.04	77	58	3
	CH_3_CH_2_CO^+^	57.03	14	4	—
	CH_3_CH_2_^+^	29.02	9	38	97
Butyric acid	CH_3_(CH_2_)_2_COOH·H^+^	89.06	71	56	4
	CH_3_(CH_2_)_2_CO^+^	71.05	11	2	—
	CH_3_CO^+^	43.02	—	4	8
	CH_3_(CH_2_)_2_^+^	43.05	10	11	3
	CH_3_CH_2_^+^	29.02	8	27	85
NO^**+**^					
Acetic acid	CH_3_CO^+^	43.02	99	97	73
	CH_3_^+^	15.02	1	3	27
Propionic acid	CH_3_CH_2_COOH^+^	74.04	95	97	33
	CH_3_CH_2_CO^+^	57.03	2	1	—
	CH_3_CH_2_^+^	29.02	3	2	67
Butyric acid	CH_3_(CH_2_)_2_COOH^+^	88.05	70	59	20
	CH_3_(CH_2_)_2_^+^	43.05	5	6	4
	CH_3_CH_2_^+^	29.02	1	5	57
	CH_3_COOH^+^	60.03	21	24	8
	CH_3_CO^+^	43.02	3	6	11
O_2_^+^					
Acetic acid	CH_3_CO^+^	43.02	93	94	79
	CH_3_^+^	15.02	—	2	18
	CH_3_COOH^+^	60.03	7	3	3
Propionic acid	CH_3_CH_2_COOH^+^	75.04	95	97	37
	CH_3_CH_2_^+^	29.02	2	3	63
	CH_3_CH_2_CO^+^	57.03	3	—	—
Butyric acid	CH_3_(CH_2_)_2_COOH^+^	88.05	37	15	9
	CH_3_COOH^+^	60.03	53	65	38
	CH_3_CO^+^	43.02	6	15	34
	CH_3_(CH_2_)_2_^+^	43.05	3	4	6
	CH_3_CH_2_^+^	29.02	1	1	13

For propionic acid (Figure 1B), the neutral loss of water (CH_3_CH_2_CO^+^; *m/z* 57.03) is already observed at low *E/N* but becomes less prominent at increasing *E/N*. The parent ion (CH_3_CH_2_COOHH^+^; *m/z* 75.04) remains the most abundant product ion up until *E/N* 150 Td. At higher *E/N*, an increased abundance of the ion at *m/z* 29.02 is recorded which corresponds to CH_3_CH_2_^+^. The product ion distribution was not affected by humid drift tube conditions.

Reactions of butyric acid with H_3_O^+^ predominantly led to the formation of CH_3_(CH_2_)_2_COOHH^+^ at low to mid-range *E/N*. Fragment ions at low *E/N* were assigned as CH_3_(CH_2_)CO^+^ (*m/z* 71.05), CH_3_(CH_2_)_2_^+^ (*m/z* 43.06), and CH_3_CH_2_^+^ (*m/z* 29.04). At higher *E/N* the CH_3_CH_2_^+^ ion became the dominant species (Figure 1C). Increased humidity in the drift tube did not change the product ion distribution.

Fragmentation of the protonated molecule substantially increased beyond *E/N* 140 Td for each SCFA. A reduced electric field of 130 Td or lower would therefore generate less complex mass spectra (fewer fragments) when using H_3_O^+^ as reagent ion. From these observations, three reaction channels could be defined in which the protonated parent molecule is formed, an acylium ion is formed due to the loss of water, and a carbocation and formic acid are formed (Supplementary Table S1).

### *E*/*N* Optimization Using NO^+^ and O_2_^+^ as Reagent Ion

The analysis of acetic acid, propionic acid, and butyric acid with different PTR-MS instrumentation has been reported previously (Hartungen et al., 2004). However, so far reactions of these SCFAs with NO^+^ and O_2_^+^ have not been investigated. The ionization energies for acetic acid, propionic acid, and butyric acid are respectively 10.65, 10.44, and 10.17 eV, which are lower than the ionization energy for O_2_ (12.1 eV), but higher than for NO (9.3 eV) (NIST, 2021). Hence, it can be expected that the ionization of SCFAs with NO^+^ will not be efficient and will subsequently lead to higher detection limits.

For each SCFA, the MH^+^ ion was observed which could result from reactions with H_3_O^+^ impurity in the drift. For both O_2_^+^ and NO^+^, the presence of H_3_O^+^ impurity was confirmed by the presence of *m/z* 19.01 (H_3_O^+^) in the mass spectrum (Supplementary Figure S2). The abundance of H_3_O^+^ was in each below 1%, which is expected (Jordan et al., 2009a). Reactions of SCFAs with NO^+^ and O_2_^+^ are listed in Table 2 and Table 3, respectively.

**TABLE 2:** Figures of merit for the analysis of acetic acid, propionic acid, and butyric acid.

Compound	Linearity			LOD (ppbV)			LOQ (ppbV)			RSD%^a^		
	H_3_O^+^	NO^+^	O_2_^+^	H_3_O^+^	NO^+^	O_2_^+^	H_3_O^+^	NO^+^	O_2_^+^	H_3_O^+^	NO^+^	O_2_^+^
Acetic acid	0.99	—	0.99	0.93	>100	1.02	2.09	>100	2.55	2.6	—	3.7
Propionic acid	0.99	—	0.99	0.34	>100	1.06	0.85	>100	2.68	3.2	—	5.7
Butyric acid	0.99	—	—	0.33	>100	49.4	0.96	>100	>100	2.5	—	—

a Relative standard deviation (RSD) measured for five replicates at 20 ppbV for each SCFA.No data for linearity and RSD, available for NO + ionization due to the high detection limit of the SCFAs.

**TABLE 3:** Repeatability of SCFAs concentrations (relative standard deviations) for five consecutive exhaled breath samples from five individuals measured with SRI/PTR-ToF-MS with H_3_O^+^ as reagent ion.

Participant	Acetic Acid (%)	Propionic Acid (%)	Butyric Acid (%)
1	13.2	7.3	9.6
2	3.0	8.9	4.8
3	8.8	2.5	7.8
4	5.6	12.5	8.8
5	2.9	6.1	8.9

Using NO^+^, no parent ion (CH_3_COOH^+^) was observed for acetic acid. This could be due to a lower ionization efficiency of NO^+^ compared to acetic acid. Selected ion flow tube—mass spectrometry (SIFT-MS) studies have shown the formation of CH_3_COOHNO^+^ (Boshier et al., 2010). This adduct ion was not observed for acetic acid when using NO^+^ as reagent ion potentially due to the different drift tube conditions used between PTR/SRI and SIFT instruments. Lowering the *E/N* might result in the formation CH_3_COOHNO^+^. Up until *E/N* 130 Td, CH_3_CO^+^ was the only product ion. At increased reduced electric fields, an increase for CH_3_^+^ was observed though CH_3_CO^+^ remained the dominant product ion (Supplementary Figure S3A). Reactions of acetic acid with O_2_^+^ did result in the formation of the parent ion CH_3_COOH^+^. The relative abundance of the parent ion steadily decreased at increasing *E/N*. As in ionization with NO^+^, the dominant product formed is CH_3_CO^+^. At higher *E/N* (>140 Td), the formation of CH_3_^+^ was also observed (Supplementary Figure S3A). The product ion distribution did not seem to be affected by humidity.

For propionic acid, the same reaction products were found using either NO^+^ or O_2_^+^. At low *E/N*, the parent ion is the dominant product. Increasing the reduced electric field led to increased formation of the CH_3_CH_2_^+^ ion. The loss of water from the parent molecule is only observed at *E/N* values below 170 Td for dry samples. Interestingly, there appears to be an effect of humidity on the product ion distribution for both ionization with NO^+^ (Supplementary Figure S3B). Under humid conditions, there is a relative increase of the parent ion up to mid-range *E/N* (*p* < 0.05). The humidity did not affect the product ion distribution of propionic acid with O_2_^+^ ionization (Supplementary Figure S4B).

Butyric acid reactions with NO^+^ and O_2_^+^ result in the formation of the same product ions (Supplementary Figures S3C, S4C). Reaction with NO + predominantly results in the formation of the parent ion (CH_3_(CH_2_)_2_COOH^+^) up until 180 Td. Beyond 180 Td, increased fragmentation on the alkyl chain resulted in the formation of CH_3_CH_2_^+^. Furthermore, the formation of CH_3_COOH^+^ is observed as a second major product ion. This product was also observed in a selected ion flow tube - mass spectrometry (SIFT-MS) study for the reaction between butyric acid and O_2_^+^ (Brůhová Michalčíková and Španěl, 2014). One should take account of this to not confuse the formation of CH_3_COOH^+^ as a butyric acid fragment to claim the presence of acetic acid. However, in the case of NO^+^, the CH_3_COOH^+^ ion is not observed for acetic acid and therefore incorrect assignment is diminished. The case for O_2_^+^ reactions is different since the main product ion is CH_3_COOH^+^ which is also observed for acetic acid. To a lesser extent, the product ion is formed at lower range *E/N* values. Higher reduced electric fields resulted again in increased fragmentation of the alkyl chain with CH_3_CH_2_^+^. being the most dominant product ion. No difference in product ion distribution was observed between dry and humid conditions. As was observed for ionization with H_3_O^+^, increased fragmentation of the most abundant was observed for *E/N* greater than 140 Td.

As for SCFA reactions with H_3_O^+^, possible reaction channels can be proposed for the drift tube reaction of SCFAs with NO^+^ and O_2_^+^ (Supplementary Table S1). The product ions consist of the parent ions, acylium ions, and carbocations. However, unraveling the full reaction mechanism during the ionization process is beyond the scope of this article.

Overall, ionization of SCFAs with NO^+^ led to a lower signal for the parent ion compared to ionization with H_3_O^+^ (Supplementary Figure S5). Hence, the ionization efficiency of SCFAs with H_3_O^+^ is higher compared to O_2_^+^ and NO^+^, which could suggest that the detection limit of SCFAs with H_3_O^+^ is lower. This would favor the use of H_3_O^+^ for exhaled breath analysis. The investigation of the detection limits for each reagent ion is further discussed in *Optimization, Validation, and Demonstration of Real-Time Breath Analysis of SCFA 343 with SRI/PTR-ToF-MS Section*.

Furthermore, the product ions were found at the same m/z for acetic acid and butyric when NO^+^ or O_2_^+^ was used as reagent ion. This may limit the confidence of identification of the SCFAs when both compounds are present in exhaled breath. Therefore, for monitoring SCFAs in exhaled breath the use of H_3_O^+^ would be recommended and SCFAs can be traced using the protonated molecule.

### Optimization, Validation, and Demonstration of Real-Time Breath Analysis of SCFA With SRI/PTR-ToF-MS

Exhaled breath analysis of SCFAs offers a great potential e.g. for real-time monitoring of dietary status. Therefore, the measurement parameters must be optimal to minimize breath-to-breath variation. To prevent condensation, the optimum temperature of the inlet line and drift tube of the instrument was found to be 110°C. The inlet flow was increased to 300 sccm to decrease the residence time in the breath sampler since the temperature of the buffer pipe is fixed at 45°C. Care should be taken that the part of the inlet line exposed to the environment (unheated) is as short as possible to minimize the temperature gradient and subsequent condensation. Also, the use of steel connectors should be avoided as SCFAs can adsorb to the surface (Kim and Kim, 2013).

Under optimized conditions, the linearity of the signal was investigated for each SCFA was performed by gas stripping on a 25 mg·L^−1^ aqueous dilution of the individual SCFAs. The measured concentrations ranged from 4 ppbV to 100 ppbV. When using NO^+^, no reaction products were observed indicating the NO^+^ is not a suitable reagent ion for the analysis of SCFAs in exhaled breath.

Excellent linearity (*R* ^2^ > 0.99) was observed for each SCFA over the investigated concentration range for ionization with H_3_O^+^ (Table 2). LODs ranged from 0.33 ppbV (butyric acid) to 0.93 ppbV (acetic acid). LOQs were found to be in the range of 0.85 ppbV (propionic acid) and 2.09 ppbV (acetic acid). Repeatability was investigated at 10 ppbV by measuring five replicates. The relative standard deviations (RSDs) ranged from 2.5% (butyric acid) and 3.2% (propionic acid) which are well within acceptable limitations (<15%) for analytical instrumentation (Hartmann et al., 1998).

For ionization with O_2_^+^, excellent linearity and detection limits were achieved for acetic acid and propionic acid. However, the LOD for butyric acid was found to be 49.4 ppbV and >100 ppbV, respectively. Therefore, further validation work was only done using H_3_O^+^ as reagent ion, since all SCFAs of interest could be detected and reproducibly measured.

**FIGURE 2:**
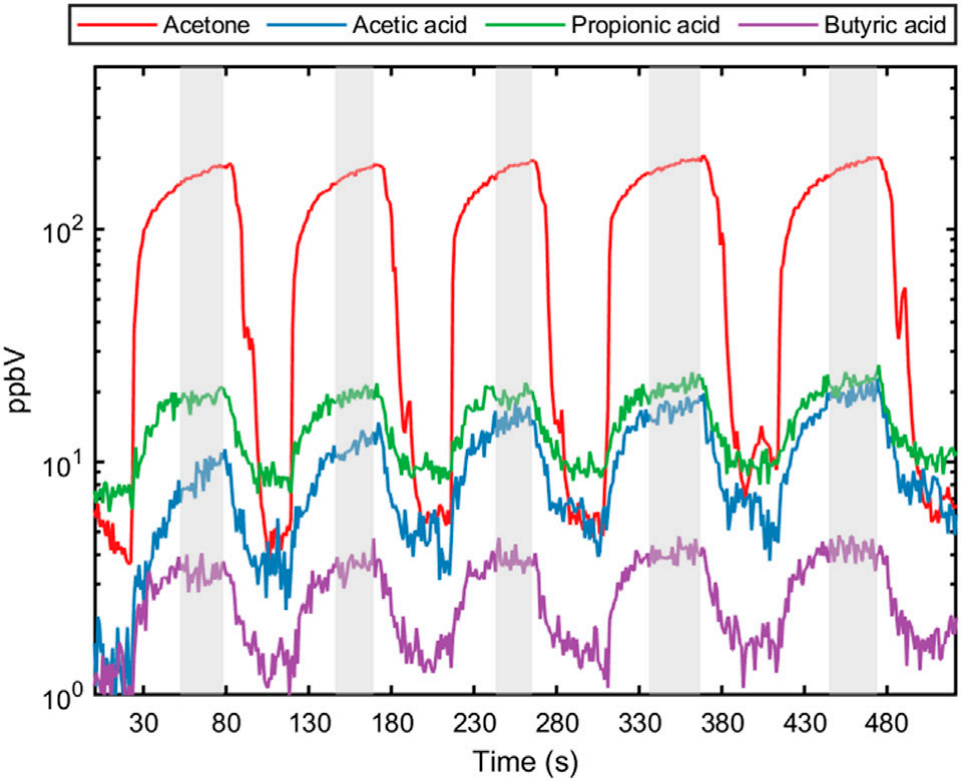
Five consecutive exhalation profiles for acetone (breath marker) and each short-chain fatty acid (acetic acid, propionic acid and butyric acid) from a healthy individual. The shaded area represents the end-tidal part of the exhaled breath used for calculating concentration and evaluating sample reproducibility.

Next, 5 consecutive exhaled breath samples were provided by each of the five healthy volunteers to validate the methodology for exhaled breath analysis. The exhalation was tracked using measured acetone as breath marker (Figure 2). A previous report on the online analysis of acetic acid using selected ion flow tube—mass spectrometry (SIFT-MS) showed poor repeatability (Lin’s concordance correlation coefficient (*R*_*c*_): 0.37) between breath replicates (Boshier et al., 2010). The repeatability of breath sampling for propionic acid and butyric acid has not been reported so far. Using SRI/PTR-ToF-MS with H_3_O^+^ as reagent ion, all SCFAs showed acceptable deviations (<15%) for analytical instrumentation (Hartmann et al., 1998) between consecutive breath samples for each volunteer (Table 3). Hence, this confirms that SCFAs can be reliably measured and quantified in real-time with SRI/PTR-ToF-MS using H_3_O^+^ as reagent ion. For demonstration, 5 exhalations were analyzed here to assess sampling repeatability, however, for studies monitoring SCFAs in exhaled breath, two exhalations would suffice. Furthermore, the effect of relative humidity was assessed on the ion intensity signal. The relative humidity of a breath sample can vary substantially (41–91%) between individuals (Mansour et al., 2020). At 50 ppbV, the normalized counts per second for the selected SCFAs were not found to be different at 100 and 50% relative humidity (*p* > 0.05).

**FIGURE 3:**
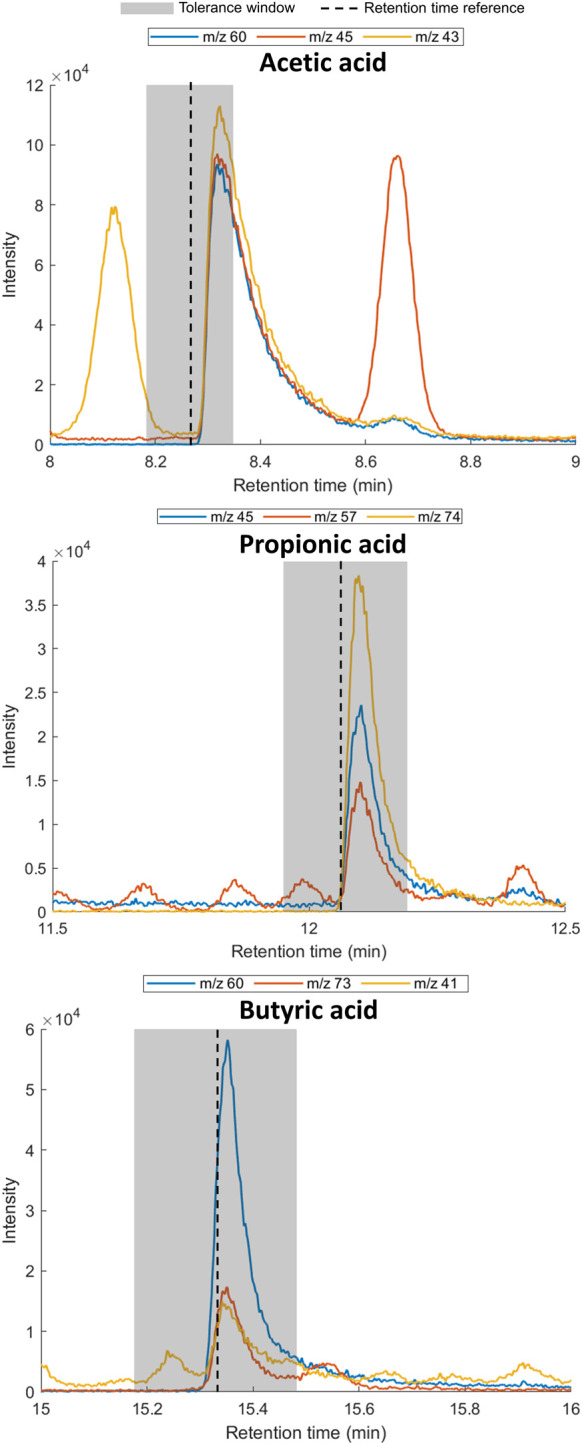
Extracted ion chromatograms for diagnostic ions for acetic acid (left), propionic acid (middle) and butyric acid (right) in exhaled breath. Shaded area indicates of 1% retention time tolerance window calculated using reference standards. The retention time of the reference standard and SCFA in exhaled breath can differ due to the effect of the matrix.

To confirm the presence of the SCFAs in the exhaled breath of the volunteer, three breath samples (1 L; deadspace discarded) were collected in a Tedlar^®^ bag and subsequently transferred to sorbent tubes (Tenax TA/Carbograph 5TD) for TD-GC-MS analysis. The presence of each SCFA was confirmed by retention time matching of the extracted ion chromatograms using at least two diagnostic ions (Table 4; Figure 3).

**TABLE 4:** GC-MS retention time data and diagnostic ions for SCFA reference standards (*n* = 3).

Compound	Retention Time	Ions (Relative Intensity %)^a^
Acetic acid	8.266 ± 0.011 min	*m/z* 60 (100), *m/z* 45 (85), *m/z* 43 (75)
Propionic acid	12.071 ± 0.010 min	*m/z* 74 (100), *m/z* 57 (38), *m/z* 45 (65)
Butyric acid	15.329 ± 0.004 min	*m/z* 73 (30), *m/z* 60 (100), *m/z* 41 (20)

a Top three ions observed in mass spectra.

Though it should be stressed that measurements were performed at systemic level and SCFAs not only originate from production in the colon. For instance, the selected SCFAs are also produced by the oral microbiota (e.g. (Gardner et al., 2019)) and can therefore contribute to the presence in exhaled breath. The concentration of SCFAs found in saliva is approximately 1–2% compared to the concentration in the colon (Bhaskaran et al., 2018). By prior rinsing of the mouth with water, the contribution of VOCs arising from the oral microbiota is minimized.

**FIGURE 4:**
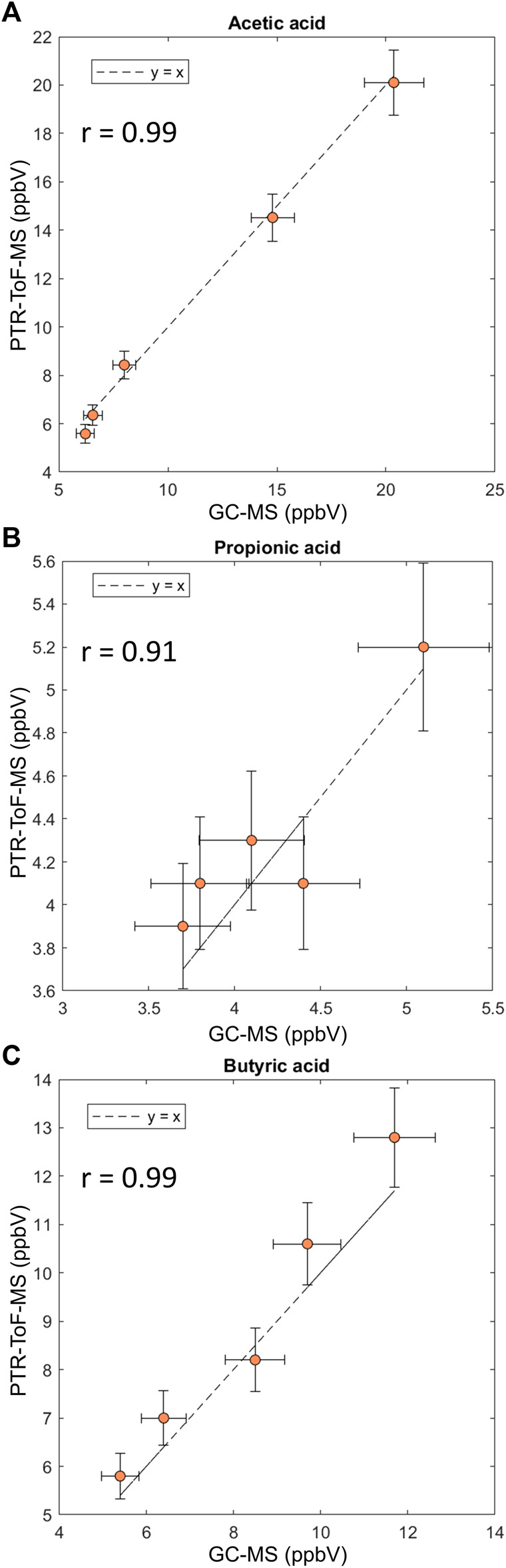
Correlation plots for comparison of quantification of SCFAs with GC-MS and PTR-ToF-MS. **(A)** Acetic acid, **(B)** propionic acid and **(C)** butyric acid. Measurements were done in triplicate. Error bars represent the standard deviation.

Concentrations found with PTR-ToF-MS were compared to TD-GC-MS results (Figure 4). For all SCFAs, an excellent correlation was found between concentrations measured with PTR-ToF-MS and TD-GC-MS indicating both methodologies are in good agreement with each other. This shows there are no interferences present in exhaled breath that affect the concentration measurement with PTR-ToF-MS.

It is known that diet affects the gut microbiome which is further influencing human health (Johnson et al., 2020). Since the interaction diet-microbiome is personalized, research is needed to understand how diet modulates the microbiome composition and how the microbiome responds to diet by producing various metabolites, including SCFA at the individual level. Also, further investigation is required into the inter-individual variation of SCFAs in healthy volunteers to establish the normal clinical range of SCFAs in exhaled breath.

## Conclusion

In this work, we optimized the conditions for SRI/PTR-ToF-MS analysis of SCFA in exhaled breath. Drift tube reactions between reagent ions and SCFAs were characterized at a wide range of reduced electric fields. Humidity did not affect the sensitivity for SCFAs. Breath sampling repeatability was found to be within acceptable limits of analytical methodology, indicating breath SCFAs can be measured with good accuracy. Also, comparison of SRI/PTR-ToF-MS measurements with the golden standard TD-GC-MS showed very good agreement. Moreover, the current strategy allows non-invasive monitoring of SCFAs, among other breath VOCs, with high sample throughput (<1 min per sample), making it an attractive alternative compared to more laborious body fluid analysis. This opens new opportunities for non-invasive monitoring of SCFAs in exhaled breath during e.g. dietary intervention studies on children.

## Data Availability

The raw data supporting the conclusion of this article will be made available by the authors, without undue reservation.
