# Supplementary material for: Real-Time Non-Invasive Monitoring of Short-Chain Fatty Acids in Exhaled Breath
Source: Front Chem. 2022 Apr 26;10:853541. doi: 10.3389/fchem.2022.853541 (PMC9285658; doi:10.3389/fchem.2022.853541)
Supplement: Supplementary file 1 [file DataSheet1.docx]

Supplementary Material


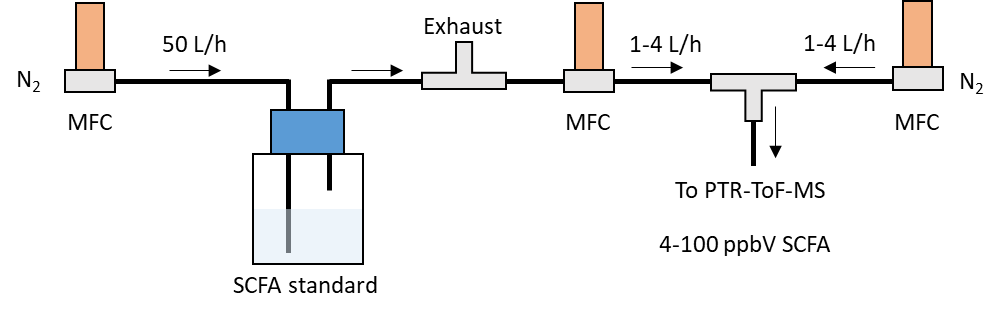


Supplementary Figure 1. Setup for generating gas-phase standards from aqueous dilutions of SCFAs by using gas stripping and mass flow controllers (MFC) to adjust the volume mixing ratio.


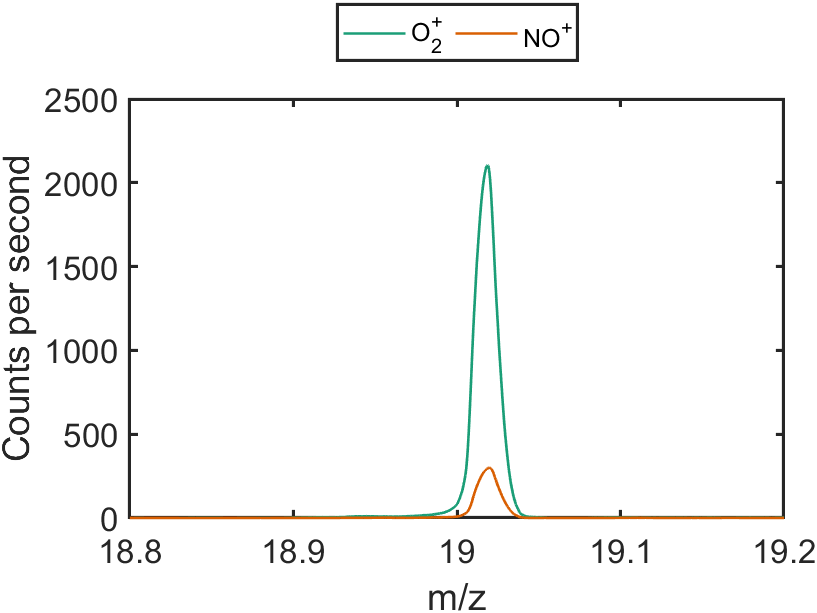


**Supplementary Figure 2.** H_3_O^+^ impurity (m/z 19.02) in the drift tube for ionization with O_2_^+^ and NO^+^ (E/N 140 Td). The percentage of H_3_O^+^ impurity was below 0.1% for both NO^+^ and O_2_^+^.

**
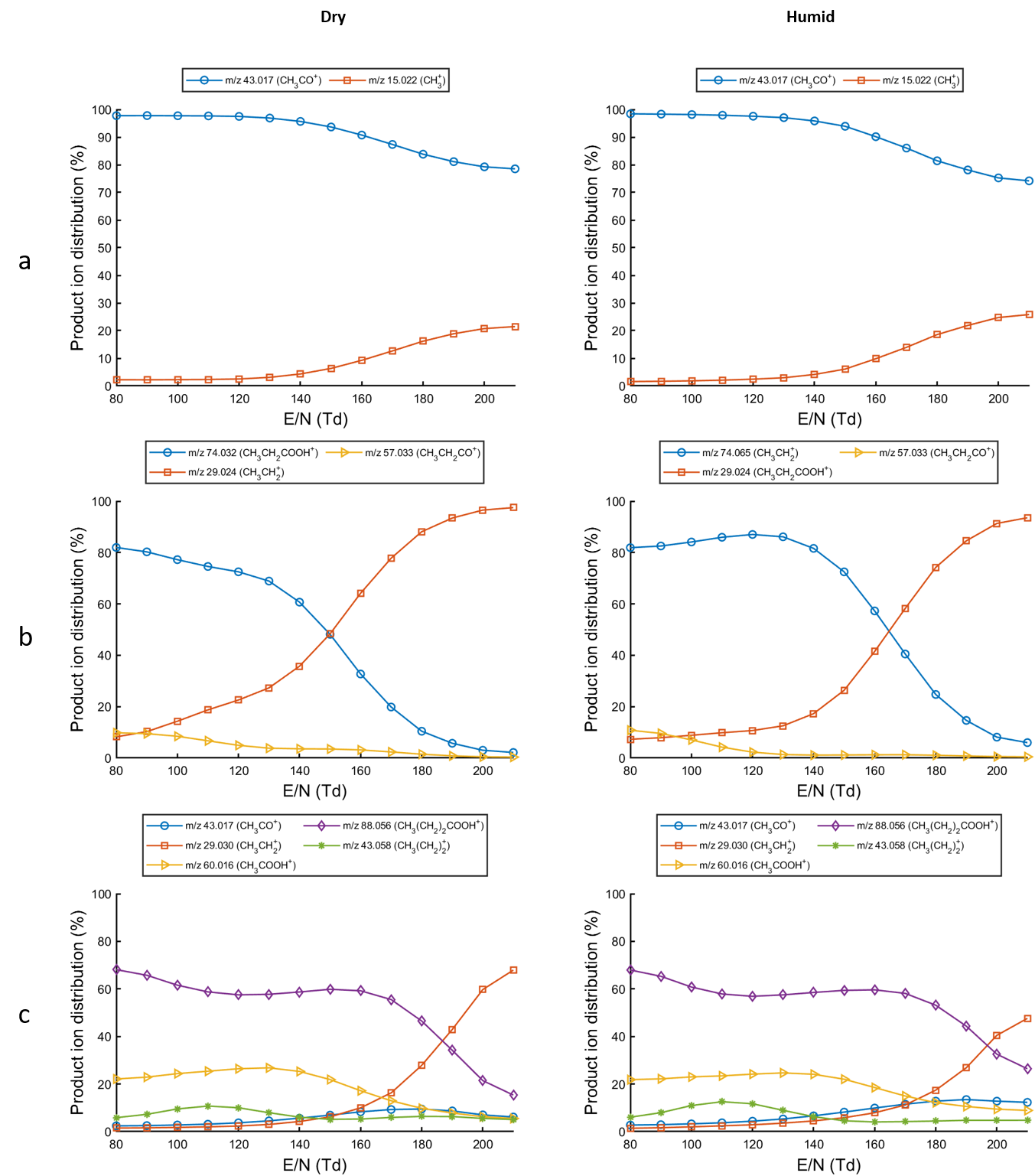
**

Supplementary Figure 3. Production ion distributions for reactions of a) acetic acid, b) propionic acid and c) butyric acid with NO^+^ in the drift tube at different reduced electric fields


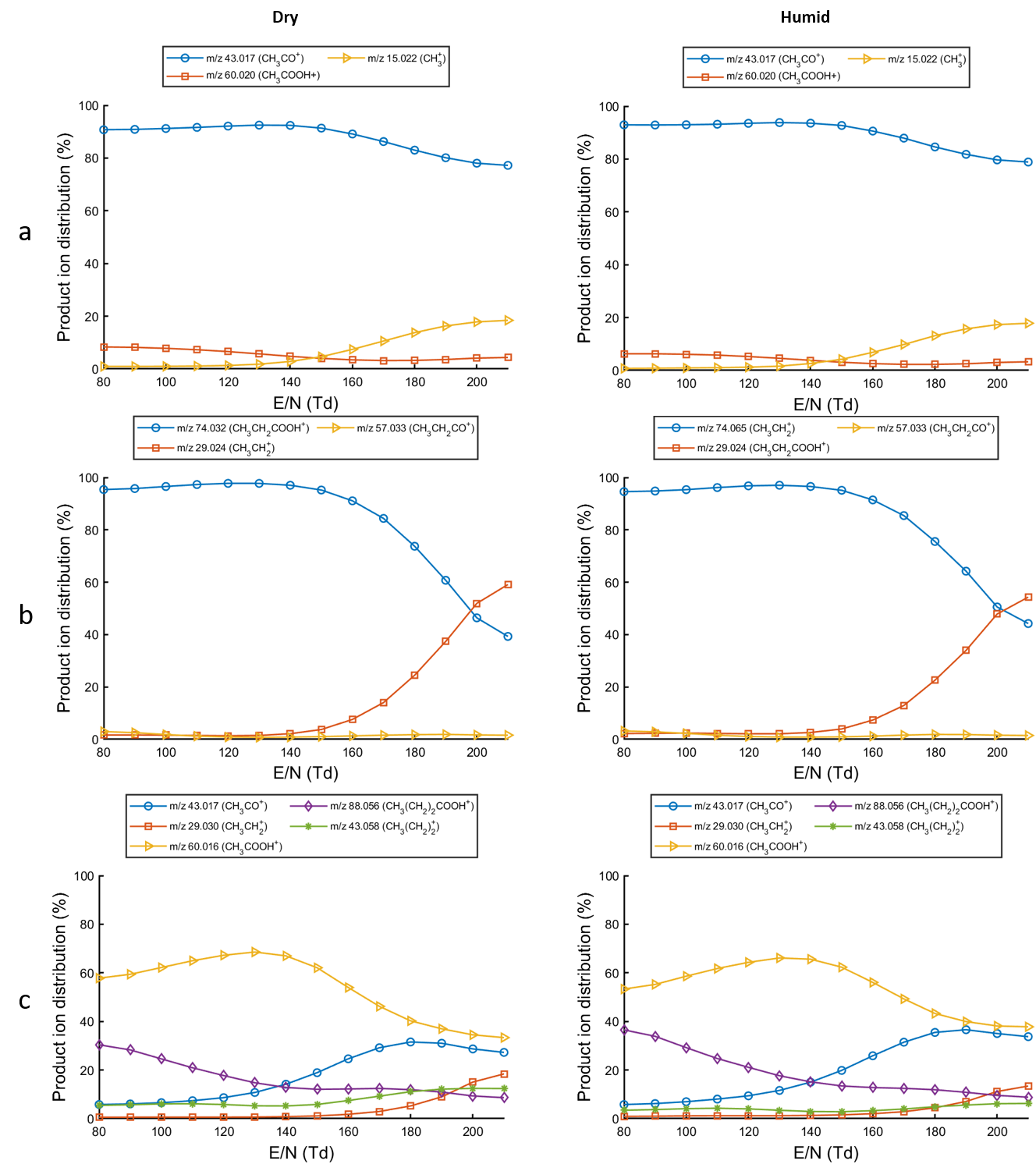


Supplementary Figure 4. Production ion distributions for reactions of a) acetic acid, b) propionic acid and c) butyric acid with O_2_^+^ in the drift tube at different reduced electric fields


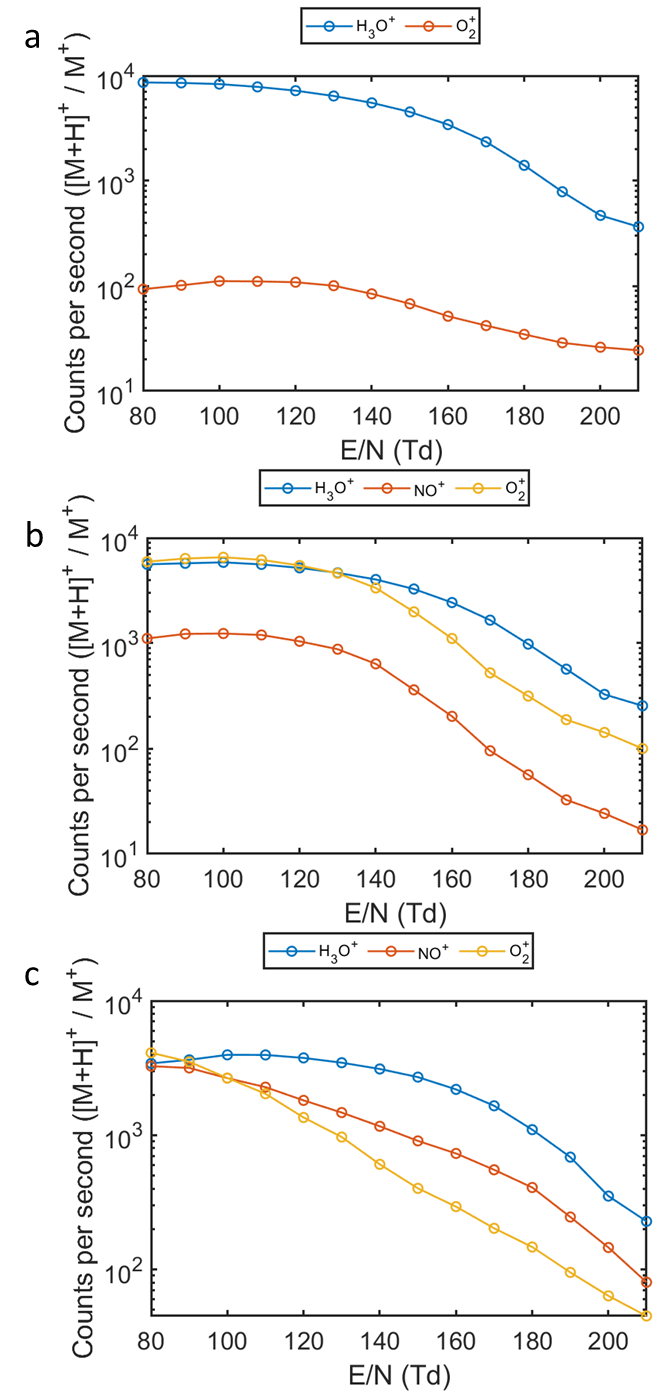


Supplementary Figure 5. Parent ion signal for a) acetic acid, b) propionic acid and c) butyric for reactions with H_3_O^+^, O_2_^+^ and NO^+^.

Supplementary Table 1. Proposed reaction channels for reactions between selected SCFAs and reagent ions

| $RCOOH+H_{3}O^{+} \underset{\to}{} RCOOH\cdot H^{+}+ H_{2}O$ | (1) |
| --- | --- |
| $RCOOH+H_{3}O^{+} \underset{\to}{} RCO^{+}+ {2 H}_{2}O$ | (2) |
| $RCOOH+H_{3}O^{+} \underset{\to}{} R^{+}+HCOOH+ H_{2}O$ | (3) |
| $RCOOH+NO^{+} \underset{\to}{} RCOOH^{+}+ NO$ | (4) |
| $RCOOH+NO^{+} \underset{\to}{} RCO^{+}+ H_{2}O+NO$ | (5) |
| $RCOOH+NO^{+} \underset{\to}{} R^{+}+HCOOH+NO$ | (6) |
| $RCOOH+{O_{2}}^{+} \underset{\to}{} RCOOH^{+}+ O_{2}$ | (7) |
| $RCOOH+O_{2}^{+} \underset{\to}{} RCO^{+}+H_{2}O+ O_{2}$ | (8) |
| $RCOOH+O_{2}^{+} \underset{\to}{} R^{+}+HCOOH+O_{2}$ | (9) |
